# Aesthetic Rehabilitation of Oligodontia in Primary Dentition with Adhesive Partial Denture

**DOI:** 10.1155/2013/872476

**Published:** 2013-11-05

**Authors:** Marília Ferreira Correia, Marianne Nicole Nogueira, Telma Blanca Bedran, Denise Madalena Palomari Spolidorio

**Affiliations:** ^1^Department of Pediatric Dentistry, Araraquara Dental School, State University of Sao Paulo, Araraquara, SP, R Humaitá 1680, 14801-903 Araraquara, SP, Brazil; ^2^Department of Diagnosis and Surgery, Araraquara Dental School, State University of Sao Paulo, Araraquara, SP, R Humaitá 1680, 14801-903 Araraquara, SP, Brazil; ^3^Department of Physiology and Pathology, Araraquara Dental School, State University of Sao Paulo, Araraquara, SP, R Humaitá 1680, 14801-903 Araraquara, SP, Brazil

## Abstract

The primary teeth are essential for bone development and establishment of the arches on occlusion. Thus, the congenitally absence of teeth may trigger a shift in the balance of the occlusion, promoting disharmony in the structures of the maxilla-mandibular system. However, some interventions are possible to be performed in these cases even in pediatric patients, to redirect growth, preventing growth deviations and reestablishing the aesthetic. The aim of this paper is to report the treatment of a 4-year-old child presenting congenitally absence of mandibular central and lateral incisors and maxilla lateral incisors, which consequently compromises aesthetics, occlusal function, and the development and the functional growth of the bones. The oral rehabilitation was performed with an adhesive partial denture, which was able to restore the aesthetic and the occlusal function, therefore being a viable alternative in the treatment of this patient of little age.

## 1. Introduction

The dental agenesis is a common developmental anomaly that affects approximately 20% of the population and results in a reduction of the number of teeth present in the oral cavity [1, 2]. Dental agenesis is classified according to the number of teeth involved and may be classified into hypodontia, oligodontia, and full anodontia. Thus, hypodontia is defined as the congenital absence of less than six permanent teeth, oligodontia as the congenital absence of more than 6 teeth and full anodontia as the absence of all permanent teeth [3]. The dental agenesis is the result of disturbances in the stages of initiation and proliferation during the formation of teeth. Its etiology is associated with environmental factors such as infections, trauma, chemotherapy, radiotherapy and genetic causes [4, 5].

The most commonly used method of, diagnosis of dental anomalies is clinical examination accompanied by radiographic examination. The periapical and the panoramic radiographs are generally used for the radiographic diagnosis of dental agenesis [6–8].

There are several treatment options for adult and young patients with agenesis, although there are few studies demonstrating treatment in pediatric patients [9]. A specialist with the patient together must make the decision regarding treatment, since it is based not only on which teeth are missing, but also on the arc length, on the position of the incisors and lips, and on the aesthetic profile. The early diagnosis and treatment are important to improve masticatory function, speech, and self-appearance to reduce the psychosocial impact [9, 10]. Thus, this paper reports the oral rehabilitation treatment of a 4-year-old patient with agenesis of six teeth in primary dentition.

## 2. Case Report

A mother of a girl of 4 years old reported to the pediatric clinic of School of Dentistry in Araraquara (State University of São Paulo) complaining about the aesthetic involvement caused by the absence of primary mandibular incisors of the child. The mother of the patient said during the anamnesis the following statement: “My daughter did not smile anymore and every time she did it, she put the hand in front of the mouth”, which demonstrated the social and mental involvement that the absence of teeth was causing.

**Figure 1:**
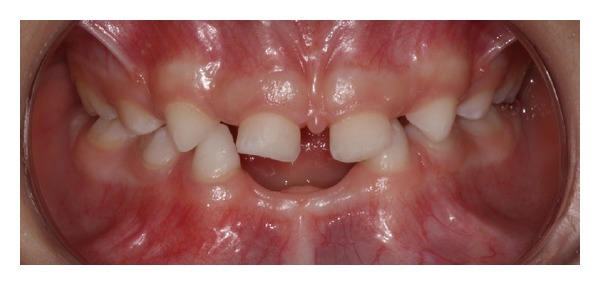
Intraoral view of dental agenesis.

**Figure 2:**
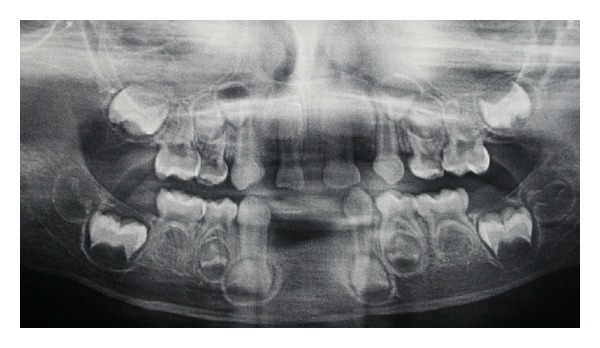
Panoramic radiograph demonstrating dental agenesis of 52, 62, 71, 72, 81, 82 (primary dentition) and 12, 22, 31, 32, 41, 42, 15, 25, 35, 45 (permanent dentition).

During clinical examination, besides the absence of mandibular incisors, the absence of upper lateral incisors was noted (Figure 1). The patient was caries-free and had no history of trauma or even any conical tooth, deciduous teeth impacted or delay in eruption of them. In view of those clinical findings, a panoramic radiograph was requested to detect the absence of permanent teeth especially. The analysis of panoramic radiograph detected the agenesis of permanent teeth as lower incisors, upper lateral incisors, and upper and lower premolars (Figure 2). Moreover, the patient had no clinical signs of any syndrome or disorder like ectodermal dysplasia or Down syndrome, which are usually syndromes related with oligodontia in primary dentition.

The treatment plan involved the installation of an adhesive partial denture for restoring only the lower incisors due to the lack of space for construction of an upper dental prosthesis (Figures 1 and 2). The choice of an adhesive prosthesis was due to the difficulty of the patient's adhesion to use a removable appliance.

**Figure 3:**
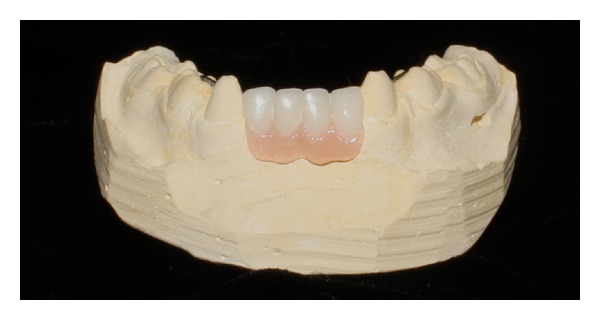
Partial dental prosthesis.

In the first appointment, the clinical procedure was carried out as follows: upper and lower molding, interocclusal record in wax, and confection of partial dental prosthesis with lower incisors. The partial prosthesis had rods made of metallic steel wire located on each side of their lateral extremity (Figure 3). These rods were designed to be fixed into the lingual surface of the canine and into the first deciduous molar lower with composite resin.

**Figure 4:**
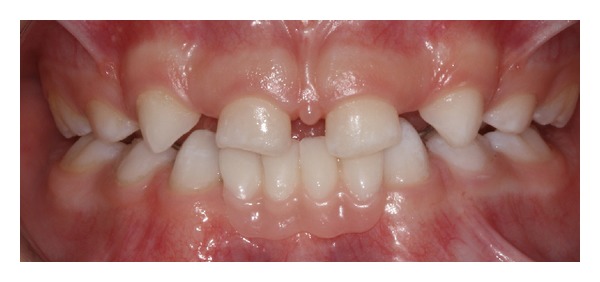
Intraoral view of oral rehabilitation.

In the second appointment, the partial prosthesis was installed as follows: etching acid of the enamel of canine and first deciduous lower molars with 35% phosphoric acid for 15 seconds, the enamel was rinsed and dried to apply only the Scotchbond Multi-purpose Plus adhesive (3 M/ESPE, USA) according to the manufacturer's directions, and finally, the rods of partial prosthesis were bonded to the surface of these teeth with a thick layer of Z250 (3 M/ESPE, USA) and the occlusion was checked.  Figure 4 demonstrated the final result of this case. Adequate care with adhesive dental prosthesis and oral hygiene was given to the mother of the patient.

## 3. Discussion

The absence of primary teeth is an uncommon condition, being present in 0.5 to 1% of the population [11]. However, when it occurs, it usually affects the upper lateral incisors or the lower incisors and is also generally associated with the absence of the permanent successor [12–14].

The etiology of dental agenesis is related to environmental factors such as rubella [15], different types of traumas to the alveolar processes [12], use of chemical substances or drugs, as thalidomide and chemotherapy [15], radiotherapy [16, 17], and disturbances in the innervation of the jaw [18, 19]. Based on current knowledge of genes and transcription factors that are involved in the development of teeth, it is assumed that different forms of dental agenesis phenotype observed clinically are caused by mutations in different genes which play distinct roles in the intracellular signaling cascade [20]. This knowledge has led us to the understanding of a wide variety of patterns of agenesis. Currently, the list of genes involved in nonsyndromic hypodontia in humans includes genes encoding transforming growth factor beta (TGF-*β*) and transcription factors (MSX1 and PAX9), which play a critical role during the craniofacial development, as well as genes encoding a protein involved in Wnt signaling pathway (AXIN2) [5, 20]. MSX1 and AXIN2 genes, involved in the early stages of odontogenesis, are associated with tooth agenesis in individuals with disorders such as cleft palate and colorectal cancer [21].

The absence of teeth is a clinical and public health problem [22], since the patients in these conditions may present several signs and symptoms as reduction of the chewing ability, malocclusion, problems in articulating words, and also the aesthetic may be compromised. These complications may affect self-esteem, behavior pattern, and social life of these patients [22, 23].

Regarding the diagnosis of oligodontia, it is normally based on radiographic evidence and routine clinical examination, detecting absence of teeth or delayed eruption of them. The panoramic radiography is the most indicated for the diagnosis and study of agenesis, due to this radiographic exam register all maxilla-mandibular regions as well as the development of the tooth germ of the patient with minimal radiation. This panoramic radiograph may be preferentially requested as soon as possible, that is, around the three and a half years old [7, 8].

The treatment goals are to keep the remaining teeth, recover the masticatory function and aesthetics, improve speech, and reestablish the emotional and psychological wellbeing [9, 11]. Regarding the prosthetic treatment, its objectives include restoring the masticatory function, maintaining the position of adjacent natural teeth preventing undesirable movements, such as inclination, extrusion, or migration, improving aesthetics, avoiding social problems to the patient, especially in adolescence, replace the missing teeth without interfering in the growth of the mandible and maxilla [24]. Considering all possibilities for oral rehabilitation of the referred patient, the prosthetic treatment of choice was the placement of adhesive partial denture due to the difficulty of the patient's adhesion to use a removable appliance. This type of prosthesis allows the restoration of function and aesthetics of missing teeth and does not interfere with bone development.

## 4. Conclusion

The congenital absence of teeth may occur as an isolated condition or may be associated with a systemic condition or syndrome clinically recognized. The clinical and radiographic examinations are the best way which the clinicians may detect anomalies in primary and permanent dentition. When cases of dental agenesis are diagnosed, treatment should be performed to restore the masticatory function and aesthetics regardless of age or missing teeth. Hence, the following case report summarized an alternative treatment of a 4-year-old patient with agenesis of six teeth in primary dentition. Besides the consequences caused by dental agenesis already mentioned, the emotional and psychological wellbeing of the child in this case was an important factor to perform an early intervention.
